# Acute Myocardial Infarction in a Patient with Twin Pregnancy: A Case Report

**DOI:** 10.5811/cpcem.2021.6.52939

**Published:** 2021-10-05

**Authors:** Tony Michaelis, Satheesh Gunaga, Tyson McKechnie, Qaiser Shafiq

**Affiliations:** * Henry Ford Wyandotte Hospital, Department of Emergency Medicine, Wyandotte, Michigan; † Henry Ford Wyandotte Hospital, Department of Medicine/Division of Cardiology, Wyandotte, Michigan

**Keywords:** Case report, acute myocardial infarction, STEMI, twin pregnancy, acute coronary syndrome

## Abstract

**Introduction:**

Acute myocardial infarction (AMI) rarely occurs during pregnancy and presents unique challenges in diagnosis and management. Traditionally, pregnancy has not readily been considered a risk factor for AMI in the emergency department despite the potential for adverse impacts on maternal and fetal health. As cardiovascular risk factors and advanced maternal age become more prevalent in society over time, the incidence will continue to increase. Prior cases with singular gestation have been reported; however, only one previous case during a twin pregnancy was identified in the medical literature.

**Case Report:**

We describe a rare case of acute ST-segment elevation myocardial infarction in a 37-year-old woman at 24 weeks gestation with a dichorionic diamniotic twin pregnancy.

**Conclusion:**

It is important for the emergency physician to recognize acute coronary syndrome as a part of the differential diagnosis of chest pain in pregnant patients and be familiar with the diagnostic and management options available for this special population.

## INTRODUCTION

It can be difficult to discern which seemingly healthy young women with no prior cardiovascular risk factors are suddenly at risk for acute myocardial infarction (AMI) simply because they are pregnant. Acute myocardial infarction has a reported incidence in pregnancy between three to 100 per 100,000 live births.^1^–^3^ Traditional cardiovascular risk factors remain pertinent in all patient populations; however, pregnancy-specific risk factors for AMI include advanced maternal age, preeclampsia, thrombophilia, postpartum infection, and hemorrhage.^3^,^4^ Physiologic changes in pregnancy contribute to increased cardiac demand, and overall the risk of ischemic cardiac events increases with maternal age.^1^,^3^,^4^ Acute myocardial infarction in pregnancy presents unique diagnostic and management challenges as seen in this case. Here we discuss an ST-segment elevation myocardial infarction (STEMI) in a 37-year-old woman at 24 weeks gestation with a dichorionic diamniotic twin pregnancy who presented to the emergency department (ED).

## CASE REPORT

A 37-year-old, gravida 4 para 3003, Caucasian female in her twenty-fourth week of pregnancy presented to the ED with intermittent bilateral arm pain of two days duration. The pain began suddenly, lasted less than 10 minutes, and resolved spontaneously without exacerbating or alleviating factors. Past medical history included hypothyroidism, anemia, and anxiety but did not include traditional cardiovascular risk factors. Her presenting blood pressure was 100/70 millimeters of mercury with a heart rate of 115 beats per minute. Physical exam was unremarkable, with normal heart sounds, no murmurs, clear lung sounds bilaterally, no peripheral edema, normal pulses, and a gravid abdomen. Initial electrocardiography showed sinus tachycardia with T-wave inversions in leads III and aVF, but no ST-segment elevations or depressions. Laboratory analysis showed a hemoglobin of 8.5 grams per deciliter (g/dL) (reference range: 12–15 g/dL) consistent with baseline anemia; otherwise, her basic metabolic, coagulation, renal, and hepatic panels were within normal limits.

**Image 1:**
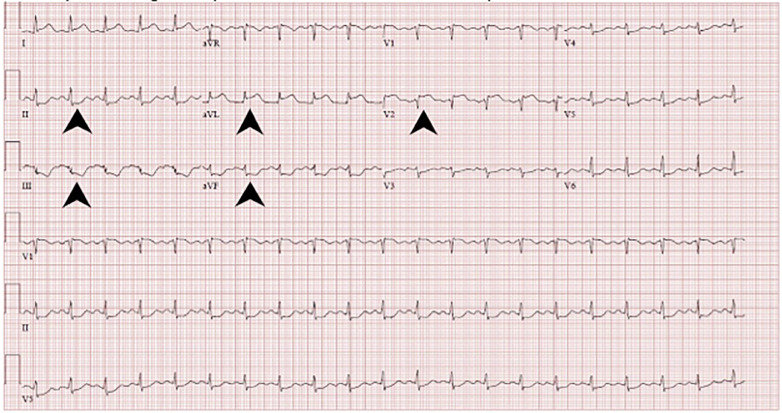
Repeat electrocardiogram one hour into emergency department visit performed during recurrence of symptoms. Black arrows on aVL and V2 show prominent ST-segment elevation, and black arrows on II, III, and aVF show reciprocal ST-segment depression. Lead I is also elevated but less prominent.

Differential diagnosis included pericarditis, myocarditis, coronary dissection, coronary vasospasm, and musculoskeletal pain. However, AMI was low on the initial differential, and troponins were not ordered on initial laboratory studies. Approximately one hour later, she experienced another episode of bilateral arm pain with radiation to her upper back. A repeat electrocardiogram was then obtained, which showed ST-segment elevations in leads I, aVL, V1 and V2 with reciprocal depressions in II, III and aVF (Image 1).

Add-on troponin-I to the initial laboratory studies was 217 nanograms per liter (ng/L) (reference range: less than 19 ng/L). Because the etiology of ST-segment changes in pregnancy can be from entities other than a ruptured atherosclerotic plaque, such as coronary dissection or vasospasm, the cardiologist and emergency physician obtained a STAT formal transthoracic echocardiogram. This showed hypokinesis of the entire anterior, anteroseptal, and anterolateral left ventricular walls and reduced ejection fraction of 40% without pericardial effusion or right ventricular enlargement (Video).

**Image 2:**
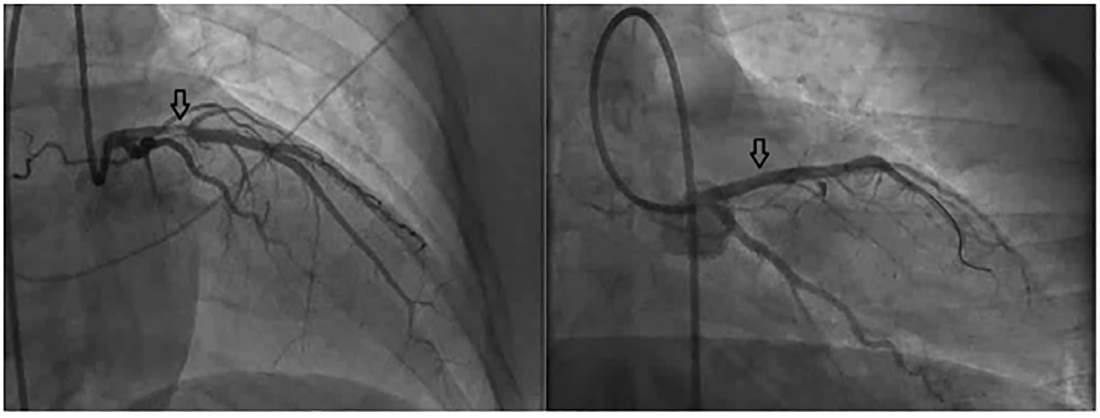
Cardiac catheterization images were taken before and after stent placement. On the left, the arrow identifies disruption of blood flow from the left anterior descending artery occlusion before stent placement. On the right, the arrow identifies the repaired left anterior descending artery after stent placement.

In the presence of significant wall motion abnormalities, AMI was highly likely, and the patient underwent emergent percutaneous coronary intervention (PCI). A repeat troponin level at two hours was 2447 ng/L. Emergent PCI revealed 90% stenosis of the proximal left anterior descending artery due to plaque rupture and thrombus, and the lesion was repaired using balloon angioplasty and placement of a drug-eluting stent (DES) (Image 2).

The patient was treated with dual antiplatelet therapy (DAPT) including aspirin and clopidogrel. She was transferred to a tertiary care hospital for high-risk maternal-fetal medicine and cardiology evaluations. Her post-PCI course was uneventful, and three days later she was discharged home. She delivered two healthy children at 35 weeks via cesarean section (C-section) that was complicated by postpartum hemorrhage. She recovered fully and remained asymptomatic on DAPT without bleeding complications on follow-up six months later.

**CPC-EM Capsule**

What do we already know about this clinical entity?

*Acute myocardial infarction (AMI) affects less than 0.1% of pregnancies, however when it occurs the lives of both mother and fetus are at significant risk of morbidity and mortality.*

What makes this presentation of disease reportable?

*Pregnancy causes physiologic changes that uniquely predispose young women towards increased risk for AMI. This is the second reported case of AMI in a twin pregnancy.*

What is the major learning point?

*Emergency medicine physicians should increase their suspicion for AMI in the pregnant population, especially in the setting of atypical symptoms.*

How might this improve emergency medicine practice?

*Early recognition of pregnancy specific risk factors and maintaining broad differentials for chest pain in pregnancy can help improve patient outcomes.*

## DISCUSSION

Acute myocardial infarction is a rare and life-threatening condition during pregnancy. Most AMIs will occur during the third trimester and peak around the peripartum and postpartum periods.^5^ Pregnant patients are a vulnerable population that may present with atypical signs and symptoms, inconsistent with classic anginal symptoms such as chest pain, shortness of breath, diaphoresis, and nausea. Symptoms may be dismissed as disorders common to pregnancy including gastroesophageal reflux, musculoskeletal complaints, and shortness of breath due to a cephalad deviation of abdominal organs.^4^ The cornerstone of emergency medicine is evaluating patients for life-threatening etiologies, especially in a pregnant patient who is at increased risk of morbidity and mortality. Although rare, AMI in pregnancy holds maternal and fetal mortality rates of up to 11% and 9%, respectively; thus, consideration of this diagnosis is critical.^2^

The etiology of STEMI in pregnancy includes atherosclerotic disease, coronary dissection, thrombosis, vasospasm of the coronary arteries, acute pulmonary embolism, and ischemia secondary to substance abuse with cocaine. One third of AMIs in pregnancy are due to underlying coronary artery disease and occur in the anterior wall involving the left anterior descending coronary artery.^6^ Our patient had none of the classic risk factors such as smoking, hypertension, obesity, family history, or diabetes, but she had pregnancy-specific risks. She was 37 years old and had an active third trimester, twin pregnancy. When compared to singleton pregnancies, multiparous gestations have a significantly higher prevalence of preeclampsia, thrombophilia, and post-partum complications, all of which are pregnancy-related risk factors for AMI.^3^,^4^,^7^ While no formal definition for advanced maternal age exists, 75% of AMI in pregnancy occurs after age 30, and 43% after age 35.^3^,^4^ Pregnant women above age 40 are more than 30 times more likely to have an AMI.^3^

Physiological changes make pregnancy a hypercoagulable state due to increased progesterone and estrogen, increasing risk of thrombosis that can cause AMI and pulmonary embolism. Other physiologic changes in pregnancy include blood volume expansion, causing increased cardiac preload, which places higher metabolic demands on the myocardium, thus increasing the risk for ischemic events. These changes contribute to a 3- to 4-fold increased risk of AMI in pregnancy.^1^,^3^ Race has not been shown to increase the risk.^3^

Electrocardiogram and troponin-I levels remain the cornerstone of AMI diagnosis. The rare occurrence of these events in combination with atypical symptoms in otherwise healthy pregnant woman often makes the diagnosis of AMI challenging. In this case, the initial ECG did not show ST-segment elevations or depressions. Acute myocardial infarction was reconsidered after her symptoms returned in the ED, which prompted the repeat ECG that showed a STEMI. Elevated troponin I is also seen in the case of pericarditis, myocarditis, demand ischemia, acute pulmonary embolism, and other cardiac conditions. However, troponins remain the standard for evaluating cardiac ischemia as levels are not affected by pregnancy, unlike creatine kinase-MB, which elevates with uterine contractions.^4^

Imaging can also be helpful in evaluating possible diagnoses and may include computed tomography and echocardiogram. We chose to obtain an echocardiogram as it is a safe, non-radiating tool that allows for maximal diagnostic return to evaluate for a pericardial effusion (dissection or pericarditis), right ventricle enlargement (pulmonary embolism), and cardiac wall motion abnormalities (AMI). Echocardiogram may also reveal pregnancy-related changes such as left ventricular hypertrophy.^2^ Computed tomography angiography for pulmonary embolism or dissection in pregnancy does not have an absolute contraindication and provides valuable diagnostic information, but risks and benefits for mother and fetus should always be considered. The American College of Obstetricians and Gynecologists recommends that life-saving interventions not be withheld from patients solely based on pregnancy.^8^,^9^

“Time is muscle” when it comes to AMI: the primary goal is reversal of the blockage as quickly as possible to preserve heart function and decrease mortality. Keys to successful treatment of an AMI include anticoagulation and emergent cardiac catheterization. Anticoagulation is a mainstay treatment for any acute coronary syndrome, excluding those caused by coronary artery and aortic dissections. Unfractionated heparin and low molecular weight heparin are preferred anticoagulation agents and are appropriate for use for AMI in pregnancy.^10^ In addition to anticoagulation, cardiac catheterization with PCI is of the utmost importance in pregnancy as a means of revascularizing the coronary arteries. The radial artery approach is preferred over femoral to minimize radiation risk and vascular damage near the fetus.^2^,^11^ Femoral access was used in our patient because of the small diameter of her radial artery and potential need of mechanical circulatory support considering she had a left anterior descending artery STEMI in twin pregnancy.

After coronary stent placement, DAPT is required to prevent stent thrombosis. For bare metal stents at least four weeks of DAPT is required, while a DES requires at least 12 months of DAPT. Studies indicate that for DES after three months, DAPT may be interrupted safely if needed. Our patient received the latest generation DES because these stents have a lower rate of in-stent restenosis and successful interruption of DAPT.^2^,^12^ Low-dose aspirin has not been shown to increase maternal or fetal mortality or cause premature closure of the ductus arteriosus, nor has it been shown to have increased risk of complications with epidural anesthesia.^10^ Given the duration and increased risk of bleeding, epidural anesthesia is contraindicated in patients receiving DAPT. Clopidogrel is identified as a class B medication in pregnancy and its use is associated with a higher incidence of C-section hemorrhages, which is likely what contributed to the postpartum hemorrhage seen in our patient on DAPT.^2^,^4^

## CONCLUSION

In summary, myocardial infarction in pregnancy is a rare but life-threatening event. This case of a twin pregnancy is even more rare, with only one prior case reported in the literature.^13^ Evaluation with imaging techniques such as an echocardiogram are of high diagnostic yield and can help guide management of STEMI in pregnancy. Early anticoagulation with low molecular weight heparin or unfractionated heparin, as well as coronary artery revascularization by PCI, are lifesaving measures that should be employed in similar case settings. Acute myocardial infarction in pregnancy is a life threat in a patient population that is generally under-recognized as being at risk. Pregnancy must be considered as a potential risk factor for cardiac disease, particularly in the face of an atypical presentation as seen in this case. The emergency physician must be vigilant in considering a broad differential diagnosis in pregnant patients and be prepared to manage this rare but potentially fatal condition.

## Supplementary Information

Video Apical four-chamber formal echocardiogram with arrows highlighting septal and apical hypokinesis. *LA*, left atrium; *LV*, left ventricle; *RA*, right atrium; *RV*, right ventricle.
